# Enhancement of Intra-hospital patient transfer in medical center hospital using discrete event system simulation

**DOI:** 10.1371/journal.pone.0282592

**Published:** 2023-04-17

**Authors:** Ekkarat Meephu, Sujitra Arwatchananukul, Nattapol Aunsri

**Affiliations:** 1 School of Information Technology, Mae Fah Luang University, Chiang Rai, Thailand; 2 Computer and Communication Engineering for Capacity Building Research Center (CCC), Mae Fah Luang University, Chiang Rai, Thailand; University of Hong Kong, HONG KONG

## Abstract

The intra-hospital transfer of critically ill patients are associated with complications at up to 70%. Numerous issues can be avoided with optimal pre-transport planning and communication. Simulation models have been demonstrated to be an effective method for modeling processes and enhancing on-time service and queue management. Discrete-event simulation (DES) models are acceptable for general hospital systems with increased variability. Herein, they are used to improve service effectiveness. A prospective observational study was conducted on 13 official day patient transfers, resulting in a total of 827 active patient transfers. Patient flow was simulated using discrete-event simulation (DES) to accurately and precisely represent real-world systems and act accordingly. Several patient transfer criteria were examined to create a more realistic simulation of patient flow. Waiting times were also measured to assess the efficiency of the patient transfer process. A simulation was conducted to identify 20 scenarios in order to discover the optimal scenario in which where the number of requests (stretchers or wheelchairs) was increased, while the number of staff was decreased to determine mean waiting times and confidence intervals. The most effective approach for decreasing waiting times involved prioritizing patients with the most severe symptoms. After a transfer process was completed, staff attended to the next transfer process without returning to base. Results show that the average waiting time was reduced by 21.78% which is significantly important for emergency cases. A significant difference was recorded between typical and recommended patient transfer processes when the number of requests increased. To decrease waiting times, the patient transfer procedure should be modified according to our proposed DES model, which can be used to analyze and design queue management systems that achieve optimal waiting times.

## Introduction

The decision to transport a patient is based on the assessment of the benefits to be gained against the potential risks. Accident and emergency care is sometimes required during patient transport, with special procedures or examinations performed outside the hospital or internal wards. Patients in critical condition may be unstable and experience changes while being moved within a hospital area. Therefore, supervision is necessary while moving patients to ensure that caregivers can prescribe medication immediately if the patient develops abnormal symptoms.

Intra-hospital patient transfer (IHT) is defined as the referral of a patient to another facility or another department within the hospital for proper diagnosis or therapeutic purposes [1]. The intra-hospital transfer of critically ill patients is associated with an overall complications rate of up to 70% [2–6]. Beckmann U et al. [2] analyzed incident reports submitted to the Australian Incident Monitoring Study in Intensive Care (AIMS-ICU) between 1993 and 1999 he found that there were severe adverse outcomes (31%), such as serious physiological derangement (15%), patient/relative dissatisfaction (7%), prolonged hospital stay (4%), physical/psychological harm (3%), and death (2%). Lovell M et al. [4] found that many complications are preventable with adequate pre-transport plans and communication. Andrews P et al. [7] found that intra-hospital transfer increased the risk of mobility and mortality relevant to operating time sequences. Gimenez FMP et al. [8] found that complications were mostly related to physiological alterations, equipment failure, team failure, and delays in transfer. Kue R et al. [9] reviewed total of 3,383 charts of patient transports within an academic quaternary-care hospital by transfer teams with special skills were correlated with low complication rates.

Previous studies focused on critically ill patient transfers. However, many intra-hospital patient transfers involve both critically and non-critically ill patients. A rise in the number of non-critically ill patients may affected the efficacy of transportation for both critically and non-critically ill patients. Over the past few years, the number of hospital patients has increased, resulting in higher demand for intra-hospital patient transfers. A super-tertiary level hospital facility in Thailand, reported 1,038,103 outpatient transfers and 212,305 inpatient transfers [10] during their 2013 annual report, which increased to 1,281,948 outpatient transfers and 337,324 inpatient transfer in 2018 [11]. Increased demand for intra-hospital patient transfers may impact the quality of this service [12]. To decrease patient transfers per episode of care may reduce the likelihood of adverse patient outcomes [13].

Recently, a computer-based online patient transfer system increased the efficiency of operations in Southern Thailand’s super tertiary hospitals [12]. This study developed a system for recording data on manually patient transfers to an online computer system, which might help manage all real-time services, including service requests, responses, assignments, and reporting on individual performance. This data can be utilized to increase work efficiency. As a result, the overall rate of on-time service delivery increased from 56.72 to 66.35 percent [12]. Key performance indicators of patient transfer in Thailand include the time taken to complete the patient transfer, within 20 minutes for outpatients, 15 minutes for inpatients, and 5 minutes for emergency cases or critically ill patients [11]. Both time and queue management for patient transport referrals must be efficient and consistent with the organizational system. The results were applied to address the issue of patient flow in the healthcare system [14, 15]. The maximum efficiency required from patient referrals is a 100% total timely service rate. The link between demand and resources was found to impact waiting time [15]. If the service capacity of patient transport is enhanced, then the accompanying costs will increase. If the service capacity is insufficient, it will increase waiting times and possibly cause patient complications. Therefore, a balance between service costs and patient transfer waiting time must be considered.

There are two types of work systems: actual and virtual [15]. Actual systems can be investigated by performing measurements, while virtual systems are conducted if the real systems cannot be quantified. An intra-hospital patient transfer could not be performed by the study during service because we were unable to make service modifications in the actual system that could cause the service to be affected directly or delayed. Computer software allows researchers to explore problems as well as examine causes and solutions using simulations that are not possible in real systems. Previously, computer models have been used to simulate the allocation of beds in intensive care units, patient flow in hospital emergency departments, patient queuing, and waiting times. Computer modeling was found to be effective in modifying variables to enhance patient service efficiency, reduce waiting times, enhance service quality, and decrease overall hazards. Simulation studies were found to have significant potential in evaluating and predicting the impact of interventions on patient flow in healthcare systems. [16–20]. In the field of computer-based simulation, various methodologies have been utilized in organizational research projects, including DES, System Dynamics, Monte Carlo, and Agent-Based Simulations [21]. Simulation model presents significant opportunities for identifying bottlenecks, determining resource requirements, and mitigating the challenges of manual planning and human error in the planning process [22]. To achieve automatic closed-loop scheduling, DES was integrated with automatic scheduling software to analyze various rules and algorithms for operations within a safe environment. The proposed model has significant potential to assist users in effectively assessing schedules and can be used for functional tests and quality assessments [23]. Healthcare simulation and modeling is a rapidly expanding area of study, and DES is a widely used stochastic modeling technique in the management of dynamic and complex systems, such as hospital management and healthcare. DES is a type of computational modeling that offers a technique that is adaptable to highly complex systems and is easy and flexible to implement [24]. However, in healthcare simulation, DES is the most commonly employed primary approach [25]. System Dynamics is less suitable for highly detailed stochastic systems. In healthcare, System Dynamics and DES have been combined to enhance healthcare simulation. However, from a philosophical perspective, it is debatable whether this is a viable approach [26].

DES is more suitable for healthcare research problems than other simulation methods, such as system dynamics, Monte Carlo, and agent-based simulations, because of its ability to accurately capture the complexity and interactions of healthcare systems. For example, DES can model the impact of changes in staffing levels, patient flow, and equipment utilization on the overall performance of a healthcare facility. Additionally, DES allows for a detailed analysis of the interactions between different departments within a healthcare facility, providing valuable insights into the functioning of the facility. For existing research, using DES to manage healthcare supply chains has long been a source of great interest [27]. The DES technique entails simulating a system’s evolution using a representation in which system state variables change instantly at discrete points in time [28]. DES method models are dynamic, discrete, and stochastic, while the technique provides a risk-free environment for testing novel ideas, policies, and decision rules that apply to a variety of scenarios [29]. The DES model can deal with dynamic and complicated systems that contain uncertainty. Simulated experiments are simple to replicate and frequently less expensive than genuine experiments. By contrast, simulation modeling involves specialized knowledge, both for model development and for analyzing and interpreting the results, which can be time-consuming as well as costly. From above discussion, DES has been widely used in modelling healthcare systems [30]. Therefore, a DES model was used to improve the service effectiveness of the general hospital system with more variability [14, 31, 32].

DES has been widely used in healthcare research to model and analyze complex systems. Several studies have reported the application of DES to the healthcare system, and when adapted to real-world scenarios, DES models have generated validated results. For example, Pan et al [33]. used DES and Design of Experiment to enhance strategies for decreasing patient turnaround time in the specialist outpatient clinic. One of the strategies with a significant impact on patient turnaround time was implemented in the actual specialist outpatient clinic system at the Singapore National Eye Centre, with encouraging results. Kuo et al [34] used a simulation model to evaluate the impact of changes in the Emergency Department and provide a tool for the operations manager to make better decisions by “foreseeing” the impact of changes like adjusting staffing levels or shift times. Kuo et al [35] created a simulation model to assess the impact of fast-track systems on waiting times for different types of patients in emergency departments. Their experiments suggest that fast-track systems can reduce overall waiting times, especially for urgent patients, and can be used to evaluate different management strategies in emergency services. Another study by Qureshi et al [36] created a DES model to predict the impact of different nurse-patient ratios. This model can assist in making operational and technical design decisions by evaluating the potential effects on nurse workload and the quality of care provided. Qureshi et al [37] used the DES model, which simulates the process of care delivery for nurses and was adapted to a real-world medical-surgical unit. They compared modeling outcomes to field-study outcomes, and the result indicates that The DES model has been validated when applied to a medical-surgical unit in practice. The DES model described in the most of published studies are unit-specific. In other words, their focus is on the resolution of specific problems in individual units of health-care systems, such as the mismatch between staffing and demand in A&E departments, the reduction of waiting times in outpatient clinics, and the increased utilization of hospital beds [30]. However, there is a scarcity of research specifically focused on intra-hospital patient transfer and the factors that impact them. This research aims to fill this gap by using DES to model and analyze intra-hospital patient transfer, and evaluate the factors that impact it. The use of DES in this research will provide valuable insights into the functioning of healthcare systems and assist in making operational and technical design decisions.

Thus, this paper presents an application of applying DES model to analyze and plan the queue management system to improve the quality of healthcare services and reduce waiting times during patient transfer. In this work we present a case study of Mae Fah Luang University which is a medical center hospital located in the city of Chiang Rai, Thailand, is presented to show the feasibility of the method.

## Materials and methods

Ethical approval for this study was obtained from the Mae Fah Luang University Ethics Committee on Human Research (COA 247/2021).

### IHT workflow and design

To minimize waiting time and improve the quality of intra-hospital patient transfer (IHT) workflow, the following simulation steps were proposed.

#### Step 1 Data collection

All parameters were collected according to the model scope for IHT analysis, including (1) distance and duration of the transfer process from the transfer center to the facility, and from facility to another facility, (2) number of requests, (3) patient status or special conditions, and (4) adverse events during the transfer process.

**Fig 1:**
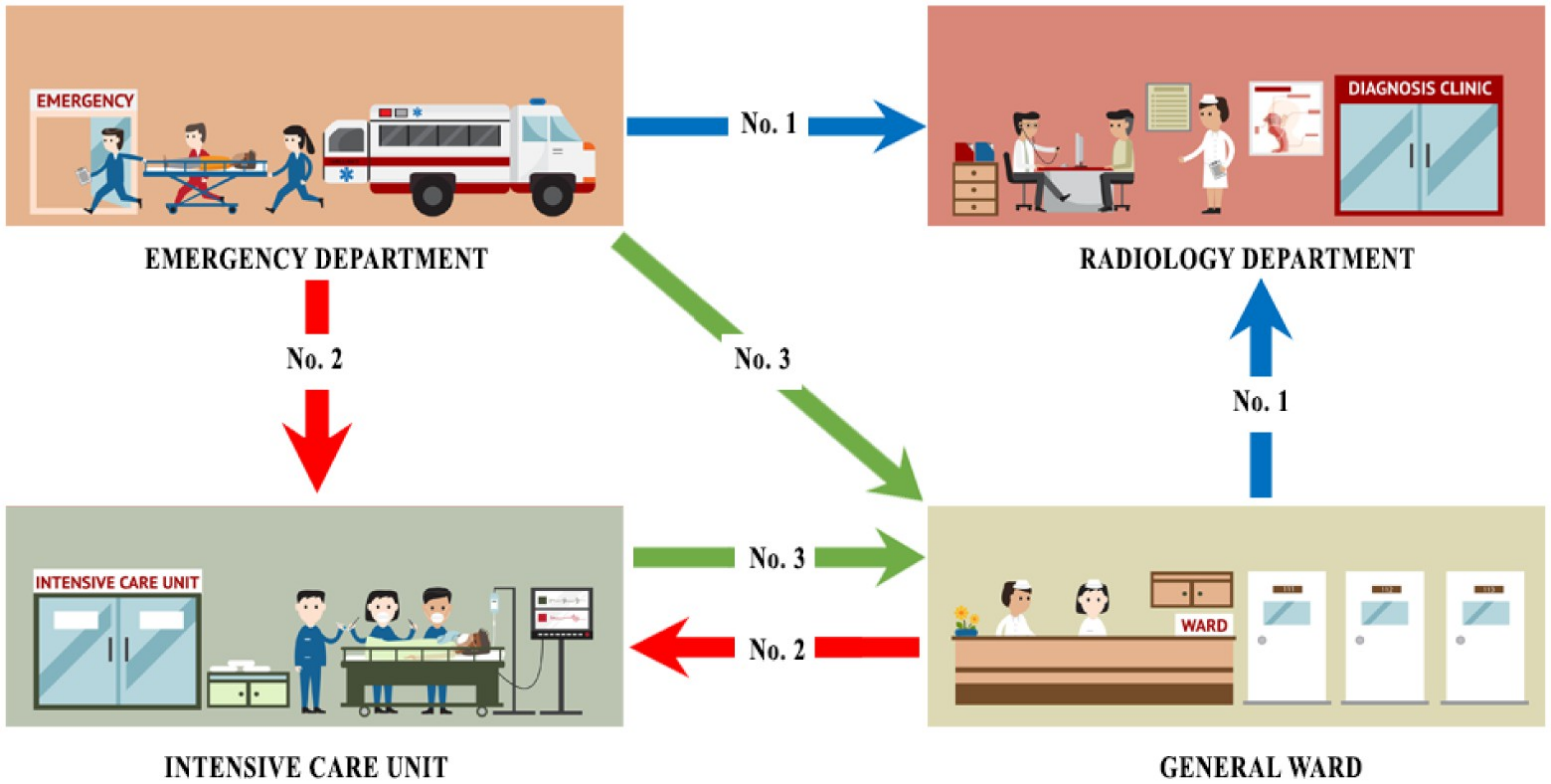
Intra-hospital patient transfer workflow from departure facility to another facility.

1. The distance was directly proportional to the time of the transfer process. We estimate distances between facilities using blueprints. The duration of the transfer procedure was utilized as a baseline for the current process, and the duration of each work was observed using a stopwatch. An example of the IHT workflow from the departure facility to another facility is shown in Fig 1 [38].
  Followings are the most usual process in intra-hospital patient transfers:
  - To move a patient who needs further examinations or advanced diagnostic procedures that cannot be performed by the current facility, e.g. from Emergency Department (ED) to Radiology Department (RD) as display by blue line in Fig 1.
  - To move a patient who needs high-intensity care to a high-intensity unit such as ED or general ward to Intensive Care Unit (ICU) or Operating Room (OR) as display by red line in Fig 1.
  - To move a patient who no longer needs close observation to continue their recovery, e.g. ICU to a general ward as display by green line in Fig 1.
2. The number of transfer requests increases the waiting time and decreases the quality of service.
3. At least two people always accompany a critically ill patient because the transfer process is potentially hazardous. These people include a nurse and a critical care technician or equivalent [39]. Patient transfer affects the allocation of staff in the organization.
4. Adverse events during the transfer process can occur with improper transfer process planning.

#### Data collection plan

Data were collected from 2–18 August 2021, as 13 official days between 00:00 and 23:59 using research assistants to observe and record the time value of every process and all collected value and submit the data in Google form.

#### Inclusion criteria

1. Serving as a hospital employee responsible for transporting patients from one department to another at Mae Fah Luang University Medical Center Hospital between August 2 and August 18, 2021
2. Agreed to take part in the study

#### Exclusion criteria for selecting samples

1. The patient transfer process may be interrupted

The following data were observed during patient transfer and separated into data sets, as shown in Table 1.

**Table 1:** Data collection for simulation modeling.

Categories	Collected Values
Period	Night, Morning, Afternoon
Department request	Drop point ER, ER, OPD, OPD-Room, X-ray, Drop point OPD, Screen point/Register, Cashier/Pharmacy, Exit, Physiotherapy, Lab Floor1, Food court, cardiac Center, OPD EENT, Cath Lab, Lab Floor3, Labor room, OB Gyne, OR, ICU, CCU, IPD9, IPD10, Consult, Refer out, Ground Floor, ER Transfer Base, OPD Transfer Base
Department Arrival	Drop point ER, ER, OPD, OPD-Room, X-ray, Drop point OPD, Screen point/Register, Cashier/Pharmacy, Exit, Physiotherapy, Lab Floor1, Food court, cardiac Center, OPD EENT, Cath Lab, Lab Floor3, Labor room, OB Gyne, OR, ICU, CCU, IPD9, IPD10, Consult, Refer out, Ground Floor, ER Transfer Base, OPD Transfer Base
Time Job request	Example: 8:30 AM
Time Departure	Example: 8:30 AM
Time Arrival	Example: 8:30 AM
Time wait for pick up (if any)	Example: 8:30 AM
Time Arrival (2) (if any)	Example: 8:30 AM
Time back to basement	Example: 8:30 AM
Staff ID	ST01- ST11
Vehicle	Wheelchair, Bed
Comment	Any
Condition	Critical ill, Non critical ill
Emergency	Emergency, Non-emergency

#### Step 2 Data analysis

Data include waiting time, number of requests, adverse events identified as bottlenecks, and optimal number of transfer staff were collected. After collecting all available data, all of patient transfer data were analyzed: Probability of incoming case severity, delivery time at each station, probability of request (occurrences per hour), vehicle, probability of station arrival, and distance between stations.

#### Step 3 Algorithm design

**Fig 2:**
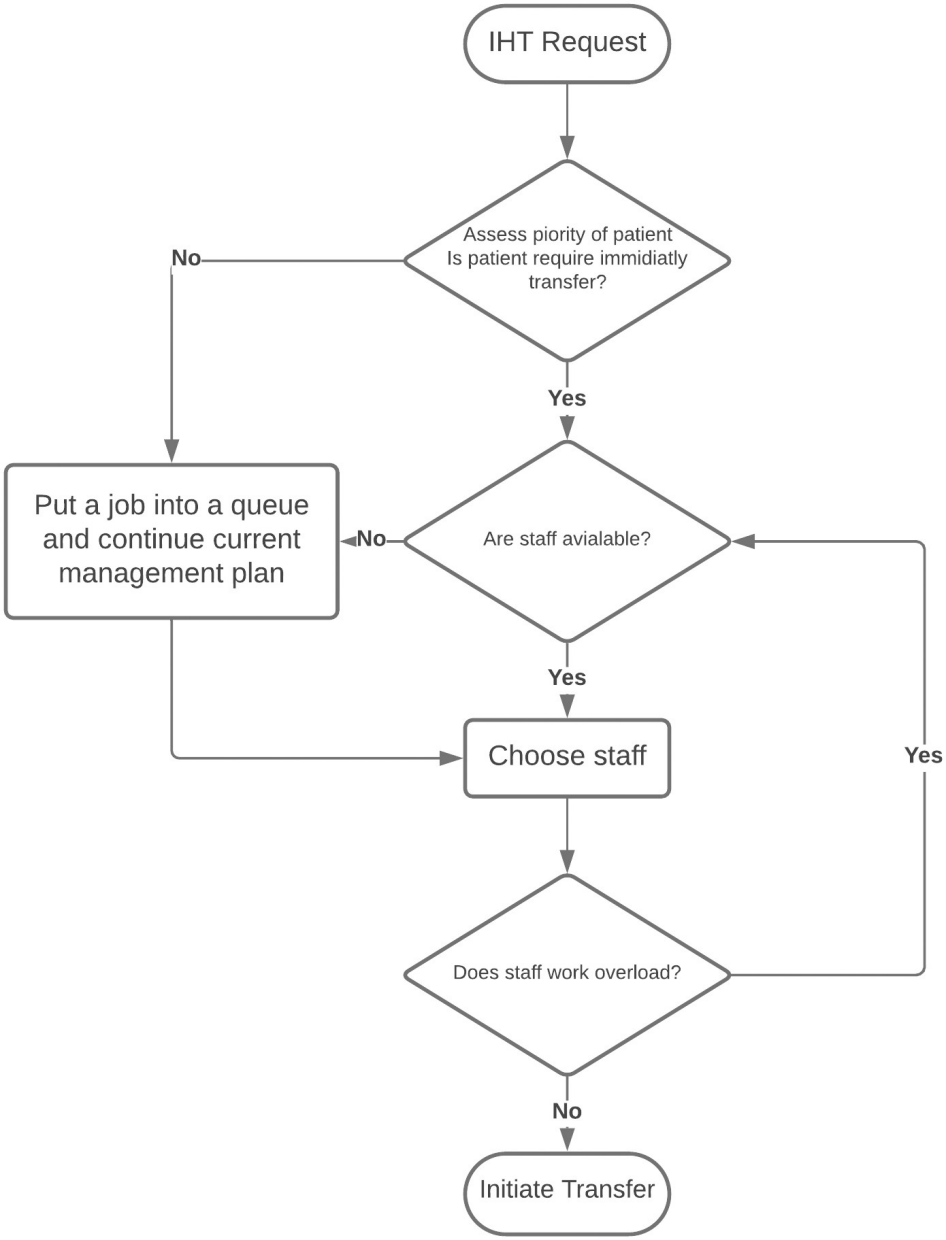
Intra-hospital patient transfer algorithm.

After evaluating the data, the ARENA^®^ simulation software was used to generate a simulation model based on the analyzed data. The model was validated as a precise and accurate representation of the current scenario. The statistical analysis was conducted by comparing the collected data to the model findings. Parameters include optimized workload allocation, reduced waiting time, and the selection of adequately experienced staff will be use to generate an IHT algorithm to minimize waiting time without increasing the number of transfer staff and select appropriate staff skills, as shown in Fig 2.

The flowchart in Fig 2 shows the procedures for the IHT algorithm, as follows:

1. The process begins when the departure facility calls the transfer center to ask for a patient transfer.
2. Patient priorities are evaluated by a doctor or nurse at the departure facility.
3. After patient evaluation, the transfer staff are selected.
4. After the staff have been selected, the system calculates the workload. If the selected staff have a significantly higher workload compared with the average workload level, the system will reselect the transfer staff. If no extra staff are available, the selected staff will be allocated regardless of their workload.
5. If no staff are available, the system will put the job event into a queue list.
6. The system will select the nearest available staff and retrieve patient information from the hospital system.
7. The process ends when the patient initiates transfer to the arrival facility.

#### Step 4 Simulation model and analysis

This step involves designing and conducting experiments to fulfill the objectives of the created scenario under various conditions such as (1) patient’s critically ill or stable condition, (2) distance between facilities, (3) number of transfer requests, (4) number of staff available, and (5) priority of the patient. The average waiting time in each situation and workload of transfer staff are collected for performance evaluation, as shown in Table 2. After validating the model, we undertake an experiment by fixing two conditions: the patient’s condition and the distance between facilities, while varying the other conditions: the number of requests (Normal = current scenario, High = increse by 20%), the number of staff (Normal = current scenario, Low = decrease by 2 staff), and the patient’s priority. Result in total 20 scenarios.

**Table 2:** Conditions for the simulation model.

Condition design for simulations	Description of intervention
**Patient condition**	Transfer of critically ill patient (e.g. severe hypertension or hypotension, patient-ventilator synchronization, severe hypoxia) with higher mortality than a non-critically ill patient.
**Distance between facilities**	Distance between facilities directly impacts the duration of the patient transfer process (e.g. distance between ER (1^st^ floor) and ICU (2^nd^ floor) is 100 meters and the elevator is a variable that impacts the duration of patient transfer, while ER (1^st^ floor) and RD (1^st^ floor) are closer than ER to ICU).
**Number of transfer requests**	An increase in the number of patients at each facility results in long patient waiting times before receiving healthcare. This reduces thequality of healthcare service, increases medical errors, and may lead to increased mobility and mortality.
**Number of available staff**	Insufficiently competent staff, especially critical care staff, (e.g. technician, paramedics) may increase waiting time, mobility, and mortality.
**Patient priority**	Number of requests for patients who require immediate transfer to advanced procedures or testing (e.g. head injury, stroke) may be interrupted by non-emergency patients as a result of inappropriate planning.

The output analysis compares the results from two or more experiments and selects the best alternative for the IHT process.

The ARENA^®^ general-purpose simulation software was chosen to develop the model. The ARENA^®^ product family consists of the ARENA^®^ Input Analyzer tool to determine probability distributions, the ARENA^®^ simulation software to identify DES-based blocks, and the ARENA^®^ Process Analyzer to select different input parameters.

**Fig 3:**
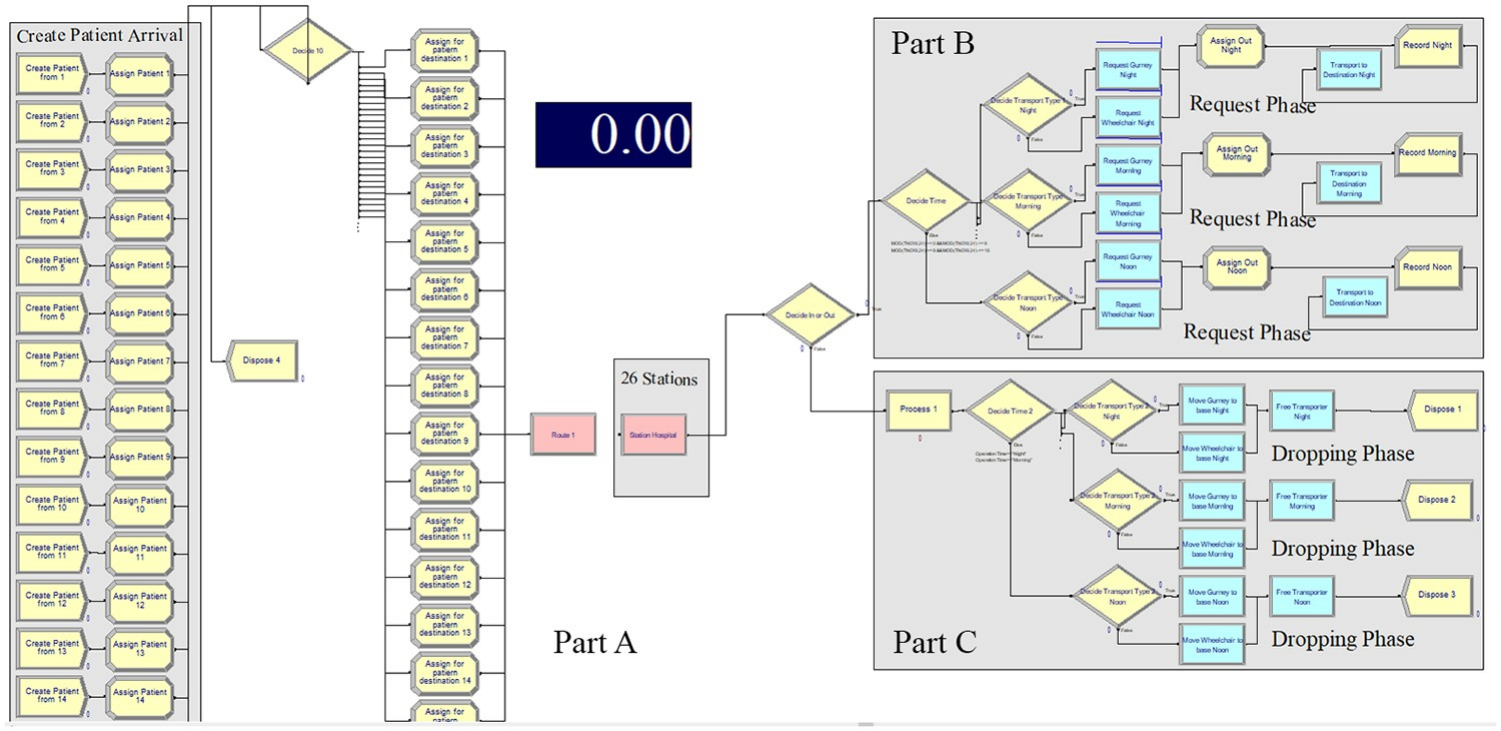
Structure of the simulation model for the intra-hospital patient transfer.

Following data collection, a discrete-event simulation (DES) model was created to analyze and plan a queue management system using ARENA^®^ simulation software version 14.00. A conceptual model flowchart is presented in Fig 3. Part A presents the structure of the simulation model for the intra-hospital patient transfer. Each station (corresponding to one row in the gray box) contains probability of patient arrival, severity, vehicle, and incoming requests from one station to the next. The total number of stations is 26 and these stations with the above quantities were used to create a model. Parts B and C respectively give more information on the request and dropping phase of each shift. Additional specifics can be found in the S1 Appendix.

The conceptual design of the model included patient arrival details. For cases where the patient required a referral process, the model established a connection with the designated available station sources. Whenever a patient requested a referral process, the model reverted to the request phase to decide on the most appropriate available transfer option.

## Results

### Data collection results

The collected data on hospital transportation indicated that patient flow identification was related to internal time of arrival, as shown in Table 3.

**Table 3:** Schedule of internal arrival times.

ID	Time Period	Time Request	Departure Time	Arrival Time	Time Finish	Dept Start	Dept Wait	Dept End	Vehicle	Staff
1	N	0:10	0:11	0:26	0:27	ER	-	IPD9	Wheelchair	ST03
2	N	0:45	0:46	0:53	0:54	ER	-	Cashier	Wheelchair	ST03
3	N	1:00	1:01	1:22	1:24	ER	-	IPD9	Wheelchair	ST05
4	N	3:21	3:22	3:24	3:25	Drop point ER	-	ER	Wheelchair	ST03
5	N	4:14	4:15	4:32	4:33	ER	X-ray	ER	Bed	ST03
6	N	5:33	5:34	5:46	5:47	ER	-	IPD9	Bed	ST03
7	M	9:34	9:35	9:37	9:38	ER	-	Exit	Wheelchair	ST01
8	M	9:37	9:38	9:40	9:41	ER	-	Exit	Wheelchair	ST01
9	M	9:42	9:43	9:45	9:46	Drop point ER	-	ER	Wheelchair	ST02
10	M	9:34	9:35	9:38	9:39	Drop point ER	-	ER	Bed	ST02
11	M	10:39	10:40	10:43	10:44	Drop point ER	-	ER	Wheelchair	ST02
12	M	10:48	10:49	10:58	10:59	ER	-	Exit	Wheelchair	ST01
13	M	11:13	11:14	11:30	11:31	ER	-	IPD10	Bed	ST02
14	M	12:23	12:27	12:38	12:39	CCU	-	Exit	Wheelchair	ST02
15	M	12:16	12:20	12:40	12:41	CCU	-	Exit	Bed	ST01
16	M	13:22	13:25	13:40	13:41	IPD9	-	Exit	Wheelchair	ST01
17	A	18:59	19:00	19:06	19:07	ER	X-ray	ER	Wheelchair	ST03
18	A	19:42	19:43	20:00	20:01	ER	-	IPD10	Wheelchair	ST02
:	:	:	:	:	:	:	:	:	:	:
993	A	23:32	23:33	23:52	23:54	ER	-	IPD9	Bed	ST03

We collected data to capture the current scenario using Google forms and automatically record patient transfer data in a spreadsheet. The ID will be automatically generated. There are three interpretations for Time Period: N = Night shift, M = Morning shift, and A = Afternoon shift. When a task was requested by phone or verbally in the case of a request from a neighboring station, a time request was recorded. When the stretcher begins transporting the patient, the departure time will be recorded. Moreover, when arriving at the location, the arrival time will be recorded. In addition, when the staff returns to the base, the time will be recorded as time finish. Call points, waiting places, patient drop-off locations, and transport vehicles are recorded. The identifying codes for personnel are recorded.

### Validation and analysis

Validation was conducted to verify the model as an accurate and precise representation of a real-world system that behaves similarly. Data were collected over 13 official days, observing day-to-day hospital patient transfer activities. Any data assumptions made during the model simulation and construction or specification of input parameters were evaluated using the ARENA^®^ simulation software Input Analyzer tool during the Data Analysis phase of this project. Table 4 displays the probability distributions of severity, operated time, and vehicle for this data. An objective analysis was required to validate Input-Output Transformations. This was accomplished by evaluating the competence of the simulation model to determine the predictive (or historical) behavior of the represented real-world systems. Using historical data, all simulation input parameters for hospital patient transfer simulation research were developed. The performance measures in Table 5, desighated as three periods of Night, Morning, and Afternoon, were used to verify the simulation model using Minitab^®^ statistical analysis software version 19.1.1 (64-bit). There were 13 official days in the real system, and 30 days of simulations were performed.

**Table 4:** Probability distributions of severity, operated time and vehicle.

station	Severity	Operated Time	Vehicle
1	0.000	-	0
2	DISC(0.804123711340206,1,1,2)	2.5 + 16 * BETA(0.571, 1.73)	DISC(0.814432989690722,1,1,2)
3	DISC(1,1,1,2)	0.5 + LOGN(5.1, 3.45)	DISC(0.96969696969697,1,1,2)
4	0.000	-	0
5	DISC(0.758333333333333,1,1,2)	1.5 + GAMM(5.78, 2.4)	DISC(0.666666666666667,1,1,2)
6	0.000	-	0
7	DISC(1,1,1,2)	1.5 + GAMM(2.4, 2.5)	DISC(1,1,1,2)
8	DISC(1,1,1,2)	1.5 + ERLA(2.97, 2)	DISC(0.981132075471698,1,1,2)
9	DISC(1,1,1,2)	0.5 + LOGN(7.62, 7.53)	DISC(0.8,1,1,2)
10	DISC(1,1,1,2)	0.5 + LOGN(6.34, 6.3)	DISC(0.964285714285714,1,1,2)
11	DISC(1,1,1,2)	NORM(11.5, 4.11)	DISC(1,1,1,2)
12	0.000	-	0
13	DISC(0.904761904761905,1,1,2)	1.5 + 17 * BETA(1.78, 1.42)	DISC(0.857142857142857,1,1,2)
14	DISC(1,1,1,2)	3.5 + WEIB(7.77, 1.85)	DISC(1,1,1,2)
15	DISC(0,1,1,2)	UNIF(7.5, 18.5)	DISC(0,1,1,2)
16	0.000	-	0
17	DISC(1,1,1,2)	5.5 + 13 * BETA(1.45, 1.25)	DISC(0.125,1,1,2)
18	DISC(1,1,1,2)	TRIA(3.5, 9, 18.5)	DISC(0.888888888888889,1,1,2)
19	DISC(0.96875,1,1,2)	POIS(15.5)	DISC(0.0625,1,1,2)
20	0.000	-	0
21	DISC(0.25,1,1,2)	POIS(10.7)	DISC(0.25,1,1,2)
22	DISC(1,1,1,2)	4.5 + ERLA(5.08, 2)	DISC(0.588235294117647,1,1,2)
23	DISC(1,1,1,2)	1.5 + WEIB(13.8, 2.74)	DISC(0.470588235294118,1,1,2)
24	DISC(1,1,1,2)	5.5 + 5 * BETA(1.34, 1.34)	DISC(1,1,1,2)
25	0.000	-	0
26	DISC(1,1,1,2)	1.5 + 4 * BETA(1.21, 1.29)	DISC(1,1,1,2)

**Table 5:** Validation result. (Hist. = historical data, Sim. = simulation model).

Period	N		Mean		SE Mean		Diff	95% CI	p-value
	Hist.	Sim.	Hist.	Sim.	Hist.	Sim.			
Night	13	30	2.31	2.07	0.82	0.27	0.241	(-1.610, 2.092)	0.784
Morning	13	30	49.54	46.63	2.5	0.94	2.91	(-2.84, 8.65)	0.298
Noon	13	30	11.77	11.33	1.4	0.63	0.44	(-2.84, 3.71)	0.781

To evaluate the validity of the model, the two-sample T-test and confidence interval (CI) were applied. The performance measure emphasized the most in terms of validating the model was the total number of patient transfers requested throughout the morning period since this reflected overall system performance. Findings of differences in mean ratings between historical data (Hist.) and the simulation model (Sim.) were 0.784, 0.298, and 0.781 for Night, Morning, and Afternoon, respectively. Because the p-value was greater than the 0.05 significance level, the null hypothesis was accepted, and the ratings of historical data and the simulation model were similar.

### Comparative analysis

The validated model was shown to be identical to the real system. This model was used to conduct simulations to determine the optimal IHT route. Table 6 shows two simulation scenarios including (i) stretcher requirements and (ii) number of staff.

**Table 6:** Simulation scenarios.

Stretcher Requirements	Normal	High	Normal	High
Number of staff	Normal	Normal	Low	Low
a. Prioritize patients with the most severe symptoms.	*a*	*a*	*a*	*a*
b. Complete the patient transfer and obtain the next one without returning to base.	*a* and *b*	*a* and *b*	*a* and *b*	*a* and *b*
c. Choose staff who are most accessible.	*a*, *b* and *c*	*a*, *b* and *c*	*a*, *b* and *c*	*a*, *b* and *c*
d. Choose the staff that have the least amount of working time to attend the job event.	*a*, *b* and *d*	*a*, *b* and *d*	*a*, *b* and *d*	*a*, *b* and *d*

In Table 6, (0) (Model “M0”) represents collected data, indicating that the need for stretchers is normal and staff = 8, while *a*, *b*, *c*, and *d* are simulations of different occurrences based on the following data. Each situation analysis was subdivided into four conditions:

1. Prioritize patients with the most severe symptoms.
2. Complete the patient transfer and begin the next transfer without returning to base.
3. Choose personnel who are more accessible.
4. Choose staff that have the least amount of working time to attend the job event.

Simulations were performed following the selection criteria as:

1. Patients with more severe symptoms must be prioritized (*a*), (Model “Ma”).
2. Prioritize patients with the most severe symptoms. After the transfer process is completed, the staff proceed to the next transfer without returning to base (*a* and *b*), (model “Mab”).
3. Prioritize patients with the most severe symptoms. After the transfer process is completed, the staff proceed to the next transfer without returning to base and choose the most accessible staff (*a*, *b*, and *c*), (model “Mabc”).
4. Prioritize patients with the most severe symptoms. After the transfer process is completed, the staff proceed to the next transfer without returning to base and choose staff that have the least amount of working time (*a*, *b*, and *d*), (model “Mabd”).

All simulations were performed for each of the four scenarios.

1. The number of stretchers required is normal and staff = 8.
2. The required number of stretchers is increased by 20% and staff = 8.
3. The number of stretchers required is normal but the staff is reduced to 6.
4. The required number of stretchers is increased by 20% and the staff is reduced to 6.

The model codes and scenarios are listed in Table 7.

**Table 7:** Model code and scenarios.

Code	Model	Stretcher Requirements	Number of staff
M0:1:6	*0*	100%	6
M0:1:8	*0*	100%	8
M0:2:6	*0*	120%	6
M0:2:8	*0*	120%	8
Ma:1:6	*a*	100%	6
Ma:1:8	*a*	100%	8
Ma:2:6	*a*	120%	6
Ma:2:8	*a*	120%	8
Mab:1:6	*a* and *b*	100%	6
Mab:1:8	*a* and *b*	100%	8
Mab:2:6	*a* and *b*	120%	6
Mab:2:8	*a* and *b*	120%	8
Mabc:1:6	*a*, *b* and *c*	100%	6
Mabc:1:8	*a*, *b* and *c*	100%	8
Mabc:2:6	*a*, *b* and *c*	120%	6
Mabc:2:8	*a*, *b* and *c*	120%	8
Mabd:1:6	*a*, *b* and *d*	100%	6
Mabd:1:8	*a*, *b* and *d*	100%	8
Mabd:2:6	*a*, *b* and *d*	120%	6
Mabd:2:8	*a*, *b* and *d*	120%	8

Each situation in the experiment was replicated for 13 official days and then repeated 30 times. As a result, model “M0:1:8” represents a typical flow; the required number of stretchers is normal and the staff number is eight.

After simulating all the possible scenarios, we obtained data on the average waiting time in each condition that was used to determine the best approach to evaluate performance.

**Fig 4:**
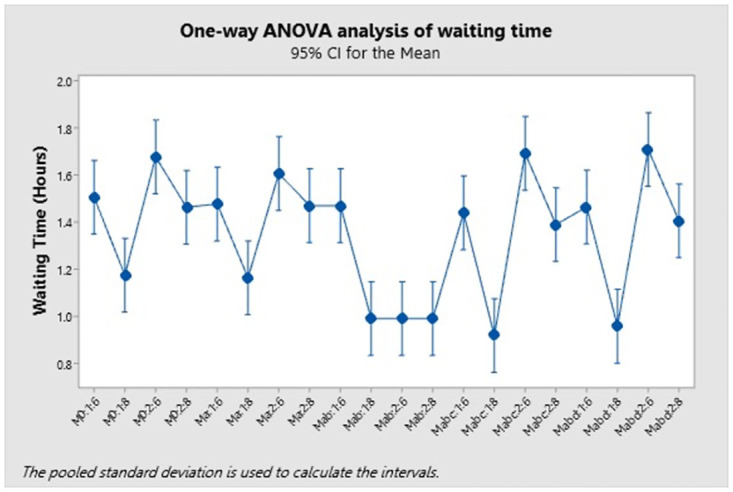
One-way ANOVA analysis of waiting time.

As shown in Table 8, “Mabc:1:8” had the lowest mean, while “Mabd:2:6” had the highest. However, we were unable to determine whether these differences were statistically significant. A one-way ANOVA analysis on the confidence intervals for mean differences was used to determine statistical significance (Fig 4).

**Table 8:** Waiting time results.

Factor	N	Mean	StDev	95% CI
M0:1:6	480	1.5048	1.9379	(1.3488, 1.6607)
M0:1:8	480	1.1751	1.6337	(1.0192, 1.3311)
M0:2:6	480	1.6752	2.1613	(1.5193, 1.8312)
M0:2:8	480	1.4624	1.9085	(1.3064, 1.6183)
Ma:1:6	480	1.4761	1.9251	(1.3202, 1.6321)
Ma:1:8	480	1.1640	1.6403	(1.0081, 1.3200)
Ma:2:6	480	1.6050	2.1241	(1.4491, 1.7610)
Ma:2:8	480	1.4691	1.9329	(1.3132, 1.6251)
Mab:1:6	480	1.4691	1.9329	(1.3132, 1.6251)
Mab:1:8	480	0.9911	1.2941	(0.8352, 1.1471)
Mab:2:6	480	0.9911	1.2941	(0.8352, 1.1471)
Mab:2:8	480	0.9911	1.2941	(0.8352, 1.1471)
Mabc:1:6	480	1.4387	1.7699	(1.2827, 1.5946)
Mabc:1:8	480	0.9193	1.2585	(0.7634, 1.0753)
Mabc:2:6	480	1.6905	1.9796	(1.5345, 1.8464)
Mabc:2:8	480	1.3890	1.6188	(1.2331, 1.5450)
Mabd:1:6	480	1.4637	1.7734	(1.3078, 1.6197)
Mabd:1:8	480	0.9590	1.2747	(0.8031, 1.1150)
Mabd:2:6	480	1.7066	1.9868	(1.5507, 1.8626)
Mabd:2:8	480	1.4050	1.6266	(1.2490, 1.5609)

Data from the simulation model indicated increases in waiting times for both scenarios covering an increase in the number of stretcher requests or a decrease in the number of staff. A suggested scenario was simulated, and the results indicated that some scenarios resulted in reduced waiting time. To determine the differences between scenarios, a one-way ANOVA analysis was performed to assess confidence intervals for mean differences. Confidence intervals are shown in Table 9.

**Table 9:** Results of mean differences.

No	Difference of Levels	Difference of Means	SE of Difference	95% CI	T-Value	Adjusted p-Value
48	Mabc:1:8—M0:2:6	-0.756	0.113	(-1.154, -0.357)	-6.72	0.000
178	Mabd:1:8—Mabc:2:6	-0.731	0.113	(-1.130, -0.333)	-6.5	0.000
52	Mabd:1:8—M0:2:6	-0.716	0.113	(-1.115, -0.318)	-6.37	0.000
106	Mabc:1:8—Ma:2:6	-0.686	0.113	(-1.084, -0.287)	-6.09	0.000
44	Mab:1:8—M0:2:6	-0.684	0.113	(-1.083, -0.286)	-6.08	0.000
181	Mabd:1:6—Mabc:2:8	0.075	0.113	(-0.324, 0.473)	0.66	1.000
107	Mabc:2:6—Ma:2:6	0.085	0.113	(-0.313, 0.484)	0.76	1.000
6	Ma:2:6—M0:1:6	0.1	0.113	(-0.298, 0.499)	0.89	1.000
111	Mabd:2:6—Ma:2:6	0.102	0.113	(-0.297, 0.500)	0.9	1.000
72	Ma:2:6—Ma:1:6	0.129	0.113	(-0.270, 0.527)	1.15	1.000

The confidence interval showed the difference between the means of group scenarios in which stretcher requirements increased and staffing levels decreased. This trend indicated that the range did not include zero, indicating that the difference between these means was statistically significant.

As shown in Table 10, the results indicated that the model can be used to optimize waiting times in scenarios with a typical number of requests and a normal number of staff as 8 by model “Mabc”, a typical number of requests and a decrease in number of staff to 6 by model “Mabc”, an increase in the number of requests to 120% and a decrease in number of staff to 6 by model “Mab”, an increase in the number of requests to 120% and a normal number of staff as 8 by model “Mab”, and an increase in the number of requests to 120% and a decrease in number of staff to 6 by model “Mab”. Adapting resources to meet patient needs is a concern in a variety of situations. In this case, the model strongly suggested that patient transfer be changed to “Mabc” and then to “Mab” if the patient number increased. For best waiting time, model “Mabd” was inapplicable because the KPI in question was waiting time, and the Model “d” selection was the lowest-performing provider. This did not benefit the waiting time, but evaluated (KPIs) others such as the quality of loading and unloading. Thus, if the objective is to quantify KPIs, the “Mabd” model is found to provide the best result. The “Mabc” model, which involved prioritizing patients with the most severe symptoms, had staff attend to the next transfer process without returning to the base after the patient transfer process was completed. Table 11 provides a more detailed analysis of the simulation results, including the Mean, Standard Deviation, and 95% Confidence Intervals of the recommended model compared to the current situation for each condition where the number of patients and staff were varied. Choosing the most accessible staff was found to be the most effective approach for decreasing waiting time in the case of the current demand number of requests and the current number of staff. The average waiting time was reduced by 21.78 percent, from 1.1751 to 0.9193 hours (70.51 minutes to 55.15 minutes).

**Table 10:** The result of the recommended model in each of the criteria.

Scenario		Management for Optimal Waiting Time
Number of Request	Number of staff	
100%	8	Mabc
100%	6	Mabc
120%	8	Mab
120%	6	Mab

**Table 11:** Detail results of simulation.

Group	Factor	N	Mean	StDev	95% CI
1	M0:1:8	480	1.1751	1.6337	(1.0192, 1.3311)
1	Mabc:1:8	480	0.9193	1.2585	(0.7634, 1.0753)
2	M0:1:6	480	1.5048	1.9379	(1.3488, 1.6607)
2	Mabc:1:6	480	1.4387	1.7699	(1.2827, 1.5946)
3	M0:2:6	480	1.6752	2.1613	(1.5193, 1.8312)
3	Mab:2:6	480	0.9911	1.2941	(0.8352, 1.1471)
4	M0:2:8	480	1.4624	1.9085	(1.3064, 1.6183)
4	Mab:2:8	480	0.9911	1.2941	(0.8352, 1.1471)

According to the results in Tables 9 and 12, model “Mabc” is capable of reducing waiting times in scenarios where demand is constant, regardless of whether the number of staff remains constant (equal to 8) or is reduced (equal to 6). When the number of staff is equal to 8, model “Mabc” is capable of decreasing average waiting time from 1.1751 to 0.9193 (difference of Means = -0.2558 (95% CI: -0.654, 0.143; p-value = 0.753). When the number of staff is reduced to 6, model “Mabc” reduces average waiting time from 1.5048 to 1.4387 (difference of Means = -0.0661 (95% CI: -0.465, 0.332; p-value = 1). Model “Mab” is capable of reducing average wait time from 1.6752 to 0.9911, a difference of means = -0.6841, when demand is increased by 120 percent and number of staff is reduced to six. (p-value: -1.083, -0.286; 95% CI: -1.083, -0.286; p-value < 0.001). When demand is increased to 120 percent and the number of staff is maintained at 8, model “Mab” is capable of reducing average waiting time from 1.4624 to 0.9911 (difference of means = -0.4712 (95% CI: -0.870, -0.073; p-value = 0.005).

**Table 12:** Result of mean difference between each of the criteria.

No	Difference of Levels	Difference of Means	SE of Difference	95% CI	Adjusted p-Value
31	Mabc:1:8—M0:1:8	-0.2558	0.113	(-0.654, 0.143)	0.753
12	Mabc:1:6—M0:1:6	-0.0661	0.113	(-0.465, 0.332)	1
45	Mab:2:6—M0:2:6	-0.6841	0.113	(-1.083, -0.286)	0
62	Mab:2:8—M0:2:8	-0.4712	0.113	(-0.870, -0.073)	0.005

## Discussion

### Study applications

This paper presents a discrete event simulation modeling framework that takes into account the complexity of an actual intrahospital patient transfer. On the basis of the collected data, it was determined that the proposed DES framework is capable of representing the performance of the current intra-hospital patient transfer. The DES model was used to propose and evaluate a set of improvement strategies, among which the factors A) Prioritize patients whose symptoms are the most severe. B) Complete the patient transfer process and immediately begin the following transfer without returning to base. C) Select personnel who are more accessible. D) Select employees who have the least amount of work time available to attend the event. Our evaluation was validated based on the outcomes of enhancing healthcare service quality and decreasing patient transfer waiting times. The robustness of Model Mabc, which entailed prioritizing patients with the most severe symptoms and personnel who complete a transfer process and begin the next transfer without returning to base, as well as selecting personnel who are more accessible. Model Mabc was demonstrated in the decrease in waiting time. This research contributes to the application of DES in healthcare management for assessing the impact of performance enhancement strategies. This paper summarizes the factors that influence the efficiency of intra-hospital patient transfers.

The results indicated that the mean difference in waiting time between the recommended and typical models was not statistically significant. However, our model significantly reduced waiting time when compared to the recommended model, even when increasing the number of patients and decreasing the number of staff. For example, the mean difference between Mabc:1:8 and M0:2:8 was -0.543 (95% CI -0.942, -0.144) (p-value < 0.001), indicating significantly reducing waiting time when increasing the number of patients by 20% and reducing staff to six. As the patient population increased, the difference in waiting times also increased.

Health services using the DES model have received considerable attention [27]. However, efforts to review and analyze previous studies have been limited due to various factors. Many modern concept classification models have been proposed to define certain developments in the implementation of DES. Some research focused solely on the usability of DES to improve staff allocation management [40]. DES should be improved in terms of usability, handling, size, and time for patient transport, whether from inside or outside of the hospital.

Despite the fact that this study was based on data collected from relatively specialized hospitals, available resources and demand were not particularly high. We propose that our findings can be applied to hospitals with comparable environments. We believe that some of the proposed solutions can be applicable to similar problems faced by other hospitals with limited staff, more complex cases, and a high demand for patient transfers.

### Study limitations

The sample size was limited by hospital location and a range of sample ages. A larger group of participants should be utilized for subsequent research projects. The scale of future experiments and the accuracy of the model should be expanded and improved as well. Due to the limits of collecting the data on other KPIs, such as delivery quality and client satisfaction, additional research is required. Examining the processes by which lower waiting times and workload reductions may be influenced other factors and have a greater effect.

## Conclusion

The discrete-event simulation (DES) model was effective for analyzing and planning a queue management system, providing significant lead times for optimal waiting time. The waiting time between each facility and station depended on factors such as distance, size of queue, and number of patients. The model improved the quality of healthcare services by reducing the waiting time of IHT via the queue management system, selecting the most severe patients for transfer on a stretcher to an empty bed, and choosing the most available staff. The model reduced mean waiting time by 21.78% from 1.1751 to 0.9193 hours (70.51 minutes to 55.15 minutes), which could be significantly important for emergency cases.

## Supporting information

S1 Appendix The specifics of the layouts and design of the simulation model for each stage are provided in S1–S3 Figs. (DOCX)

## References

[1] Nakayama DK, Lester SS, Rich DR, Weidner BC, Glenn JB, Shaker IJ. Quality improvement and patient care checklists in intrahospital transfers involving pediatric surgery patients. Journal of Pediatric Surgery. 2012;47(1):112–118. doi: 10.1016/j.jpedsurg.2011.10.030 22244402

[2] Beckmann U, Gillies DM, Berenholtz SM, Wu AW, Pronovost P. Incidents relating to the intra-hospital transfer of critically ill patients. Intensive Care Medicine. 2004;30(8):1579–1585. 10.1007/s00134-004-2177-9 14991102

[3] Gillman L, Leslie G, Williams T, Fawcett K, Bell R, McGibbon V. Adverse events experienced while transferring the critically ill patient from the emergency department to the intensive care unit. Emergency Medicine Journal. 2006;23(11):858–861. doi: 10.1136/emj.2006.037697 17057138 PMC2464383

[4] Lovell M, Mudaliar M, Klineberg P. Intrahospital transport of critically ill patients: complications and difficulties. Anaesthesia and intensive care. 2001;29(4):400–405. doi: 10.1177/0310057X0102900412 11512652

[5] Waydhas C, Schneck G, Duswald K. Deterioration of respiratory function after intra-hospital transport of critically ill surgical patients. Intensive care medicine. 1995;21(10):784–789. doi: 10.1007/BF01700959 8557864

[6] Waydhas C. Equipment review: Intrahospital transport of critically ill patients. Critical Care. 1999;3(5):1–7. doi: 10.1186/cc362 11094486 PMC137237

[7] Andrews P, Piper I, Dearden N, Miller J. Secondary insults during intrahospital transport of head-injured patients. The Lancet. 1990;335(8685):327–330. doi: 10.1016/0140-6736(90)90614-B 1967776

[8] Gimenez FMP, de Camargo WHB, Gomes ACB, Nihei TS, Andrade MWM, Valverde MLdAFS, et al. Analysis of Adverse Events during Intrahospital Transportation of Critically Ill Patients. Critical care research and practice. 2017;2017:6847124–6847124. doi: 10.1155/2017/6847124 29062574 PMC5618745

[9] Kue R, Brown P, Ness C, Scheulen J. Adverse Clinical Events During Intrahospital Transport by a Specialized Team: A Preliminary Report. American Journal of Critical Care. 2011;20(2):153–162. doi: 10.4037/ajcc2011478 21362719 PMC3715047

[10] Division SHPT. Annual Reports Siriraj Hospital, Mahidol University; 2013. Available from: https://www.si.mahidol.ac.th/th/annual-report2013/files/246.pdf.

[11] Division SHPT. Annual Reports Siriraj Hospital, Mahidol University; 2018. Available from: https://www.si.mahidol.ac.th/annualreport/2018/PDF/50.pdf.

[12] Jaroon K. Efficiency Improvement of Patients’ Transportation at the Super Tertiary Hospital in Southern Thailand. Nursing Journal of The Ministry of Public Health. 2017;27:104–120.

[13] Blay N, Roche M, Duffield C, Xu X. Intrahospital transfers and adverse patient outcomes: An analysis of administrative health data. J Clin Nurs. 2017 Dec;26(23-24):4927–4935. doi: 10.1111/jocn.13976 28748563

[14] Hall R, Belson D, Murali P, Dessouky M. In: Modeling patient flows through the healthcare system. Springer; 2006. p. 1–44.

[15] Hillier F, Lieberman G. Introduction to operations research. McGraw-Hill; 2015.

[16] Seung-Chul K, Ira H, Karl KY, Thomas AB. Flexible bed allocation and performance in the intensive care unit. Journal of Operations Management. 2000;18(4):427–443. doi: 10.1016/S0272-6963(00)00027-9

[17] Su S, Shih CL. Modeling an emergency medical services system using computer simulation. International Journal of Medical Informatics. 2003;72(1):57–72. doi: 10.1016/j.ijmedinf.2003.08.003 14644307

[18] Wang Q. Modeling and analysis of high risk patient queues. European Journal of Operational Research. 2004;155(2):502–515. doi: 10.1016/S0377-2217(02)00916-5

[19] Patrick J, Puterman ML. Reducing Wait Times through Operations Research: Optimizing the Use of Surge Capacity. Healthc Policy. 2008;3(3):75–88. 19305770 PMC2645141

[20] Vanbrabant L, Braekers K, Ramaekers K, Nieuwenhuyse I. Simulation of emergency department operations: A comprehensive review of KPIs and operational improvements. Computers & Industrial Engineering. 2019;131:356–81 doi: 10.1016/j.cie.2019.03.025

[21] Alfa AS. Queueing theory for telecommunications: discrete time modelling of a single node system. Springer Science & Business Media; 2010.

[22] Herbert M, Selmaier A, Mühlmann F, Franke J. Application of discrete-event simulation for factory planning—A case study. Simulation in Produktion und Logistik. 2021:133:15–17.

[23] Skawina B, Astrand M, Sundqvist F, Greberg J, Salama A, Ekbeck P. Automatic closed-loop scheduling in underground mining using discrete event simulation. Journal of the Southern African Institute of Mining and Metallurgy. 2021:121(6):277–282. doi: 10.17159/2411-9717/679/2021

[24] Karnon J, Stahl J, Brennan A, Caro JJ, Mar J, Möller J. Modeling Good Research Practices Task Force. Modeling using discrete event simulation: a report of the ISPOR-SMDM Modeling Good Research Practices Task Force–4. Medical decision making. 2012:32(5):701–711. doi: 10.1177/0272989X12455462 22990085

[25] Brailsford SC, Harper PR, Patel B, Pitt M. An analysis of the academic literature on simulation and modelling in health care. Journal of simulation. 2009:3(3):130–140. doi: 10.1057/jos.2009.10

[26] Brailsford SC, Desai SM, Viana J. Towards the holy grail: combining system dynamics and discrete-event simulation in healthcare. Proceedings of the 2010 winter simulation conference. IEEE, 2010:2293–2303.

[27] Kammoun A, Loukil T, Hachicha W. The use of discrete event simulation in hospital supply chain management. In: 2014 International Conference on Advanced Logistics and Transport (ICALT); 2014. p. 143–148.

[28] Law AM, Kelton WD. Simulation modeling and analysis. McGraw-Hill series in industrial engineering and management science. Boston: McGraw-Hill, c2000 3rd ed.; 2000.

[29] Ma J, Caldecott B, Volz U. Case Studies of Environmental Risk Analysis Methodologies. 2020.

[30] Günal M M, Pidd M. Discrete event simulation for performance modelling in health care: a review of the literature. Journal of Simulation. 2010;4(1):42–51 doi: 10.1057/jos.2009.25

[31] Hu X, Barnes S, Golden B. Applying queueing theory to the study of emergency department. operations: a survey and a discussion of comparable simulation studies. International Transactions in Operational Research. 2018;25(1):7–49. doi: 10.1111/itor.12400

[32] Laker LF, Torabi E, France DJ, Froehle CM, Goldlust EJ, Hoot NR, et al. Understanding Emergency Care Delivery Through Computer Simulation Modeling. Academic Emergency Medicine. 2018;25(2):116–127. doi: 10.1111/acem.13272 28796433 PMC5805575

[33] Pan C, Zhang D, Kon AW, Wai CS, Ang WB. Patient flow improvement for an ophthalmic specialist outpatient clinic with aid of discrete event simulation and design of experiment. Health Care Manag Sci. 2015;18(2):137–55. doi: 10.1007/s10729-014-9291-1 25012400

[34] Kuo YH, Rado O, Lupia B, Leung J, Graham C. Improving the efficiency of a hospital emergency department: a simulation study with indirectly imputed service-time distributions. Flexible Services and Manufacturing Journal. 2016;28(1):120–147. doi: 10.1007/s10696-014-9198-7

[35] Kuo YH, Leung J, Graham C, Tsoi K, Meng H. Using simulation to assess the impacts of the adoption of a fast-track system for hospital emergency services. Journal of Advanced Mechanical Design, Systems, and Manufacturing. 2018;12(3):JAMDSM0073–JAMDSM0073 doi: 10.1299/jamdsm.2018jamdsm0073

[36] Qureshi SM, Purdy N, Mohani A, Neumann WP. Predicting the effect of Nurse‐Patient ratio on Nurse Workload and Care Quality using Discrete Event Simulation. Journal of Nursing Management. 2019;27(5):971–980. doi: 10.1111/jonm.12757 30739381

[37] Qureshi SM, Purdy N, Neumann WP. Developing a modelling approach to quantify quality of care and nurse workload—Field validation study. Operations Research for Health Care. 2021;29:100301. doi: 10.1016/j.orhc.2021.100301

[38] Meephu E, Arwatchananukul S, Aunsri N. A Framework for Development of an Intra-Hospital Patient Transfer Using Queue Management System. In: 2018 Global Wireless Summit (GWS);. p. 300–303.

[39] Warren J, Fromm RE Jr, Orr RA, Rotello LC, Horst HM, Medicine ACoCC. Guidelines for the inter-and intrahospital transport of critically ill patients. Critical care medicine. 2004;32(1):256–262. doi: 10.1097/01.CCM.0000104917.39204.0A 14707589

[40] Hamrock E, Paige K, Parks J, Scheulen J, Levin S. Discrete Event Simulation for Healthcare Organizations: A Tool for Decision Making. Journal of Healthcare Management. 2013;58(2). doi: 10.1097/00115514-201303000-00007 23650696

