# Supplementary material for: Enhancement of Intra-hospital patient transfer in medical center hospital using discrete event system simulation
Source: PLoS One. 2023 Apr 17;18(4):e0282592. doi: 10.1371/journal.pone.0282592 (PMC10109477; doi:10.1371/journal.pone.0282592)

**Supporting information**

S1 Appendix.

Figure 1. Detail of the simulation model pertaining to the arrival phase of patients.
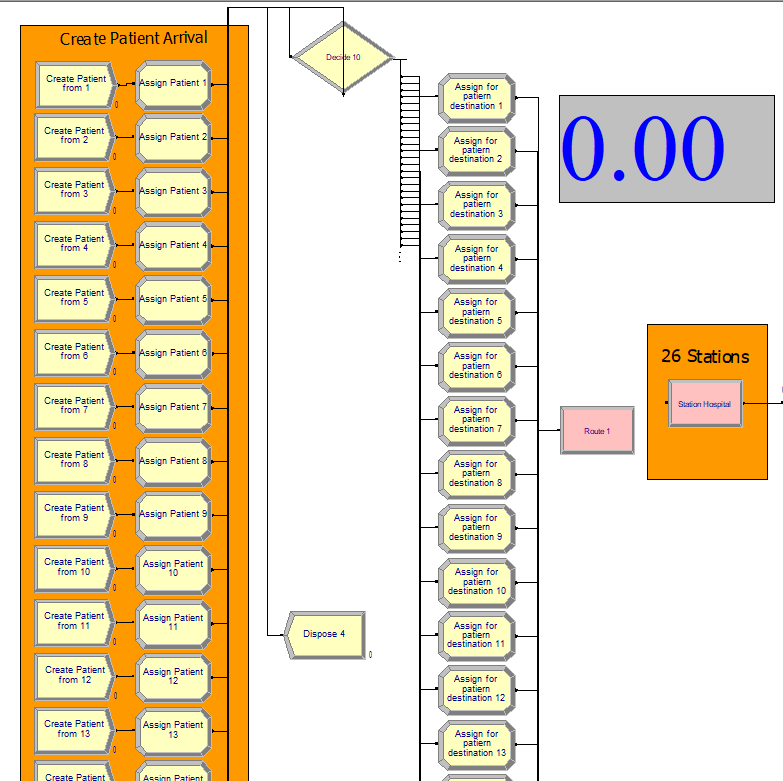


Figure 2. Detail of the simulation model regarding the request phase.


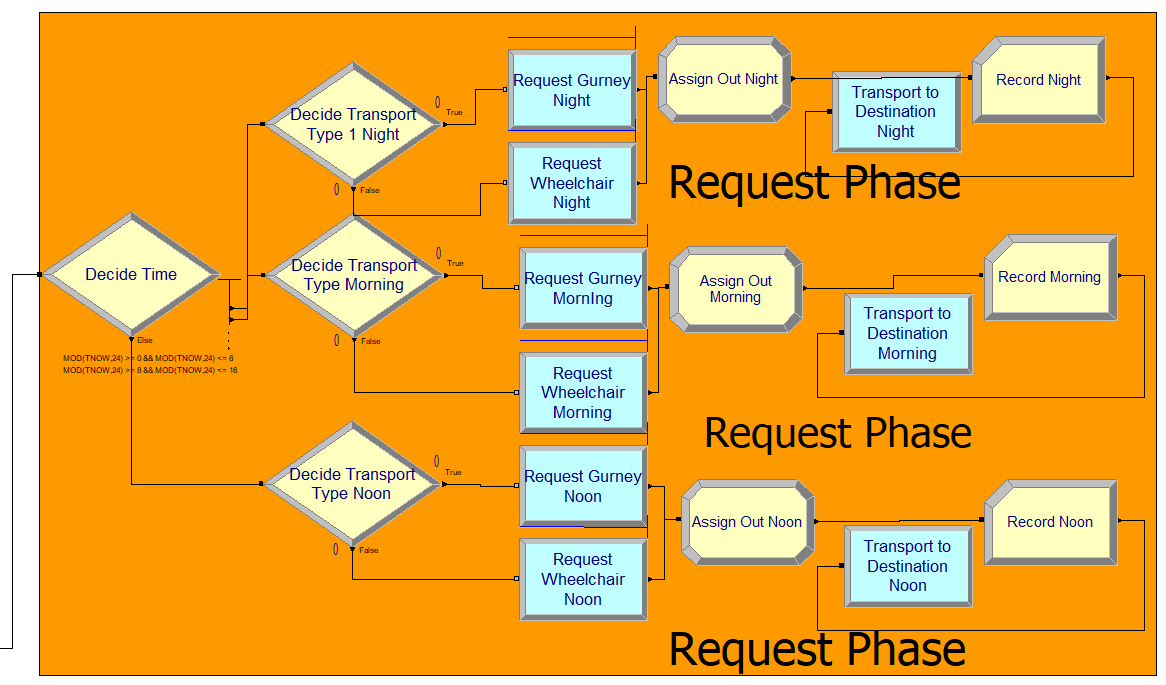


Figure 3. Detail of the simulation model regarding the dropping phase.


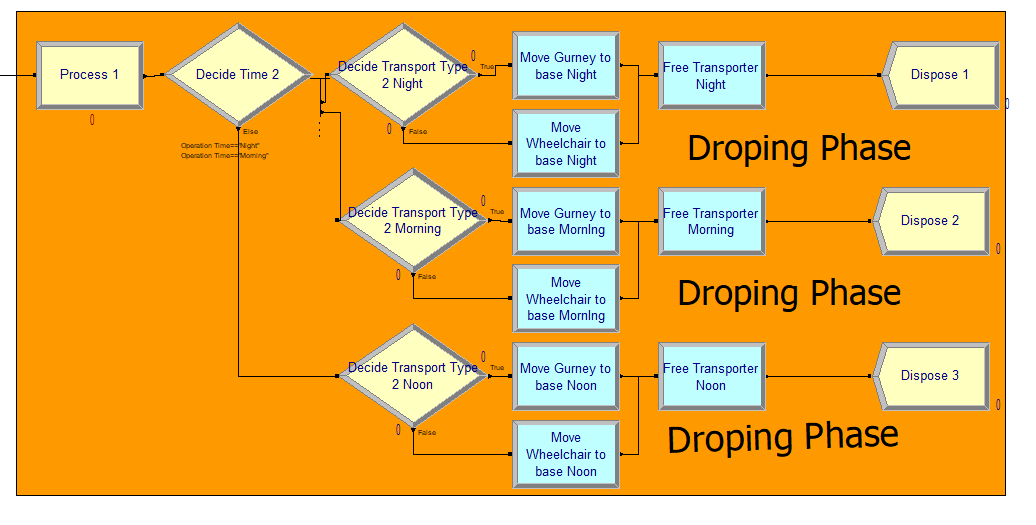

Supplement: S1 Appendix — (DOCX) [file pone.0282592.s001.docx]
